# Elective Cardiac Procedure Patients Have Low Preoperative Cardiorespiratory Fitness

**DOI:** 10.1055/a-2161-4137

**Published:** 2023-10-06

**Authors:** Sini Vasankari, Visa Mahlamäki, Juha Hartikainen, Ville Vasankari, Kari Tokola, Henri Vähä-Ypyä, Vesa Anttila, Pauliina Husu, Harri Sievänen, Tommi Vasankari, Jari Halonen

**Affiliations:** 1 Clinical Medicine, University of Turku Faculty of Medicine, Turku, Finland; 2 Heart Center, Kuopio University Hospital, Kuopio, Finland; 3 Clinical Medicine, University of Eastern Finland – Kuopio Campus, Kuopio, Finland; 4 Neurosurgery, Helsinki University Central Hospital, Helsinki, Finland; 5 UKK Institute, UKK Institute, Tampere, Finland; 6 Heart Center, TYKS Turku University Hospital, Turku, Finland; 7 Faculty of Medicine and Health Technology, Tampere University, Tampere, Finland

**Keywords:** aortic valve disease, coronary artery disease, mitral valve insufficiency, physical activity

## Abstract

Preoperative cardiorespiratory fitness may influence the recovery after cardiac procedure. The aim of this study was to investigate the cardiorespiratory fitness of patients scheduled for elective cardiac procedures, using a six-minute walk test, and compare the results with a population-based sample of Finnish adults. Patients (n=234) awaiting percutaneous coronary intervention or coronary angiography, coronary artery bypass grafting, aortic valve replacement or mitral valve surgery performed the six-minute walk test. VO _2_ max was calculated based on the walk test. The patients were compared to a population-based sample of 60–69-year-old Finnish adults from the FinFit2017 study. The mean six-minute walk test distances (meters) and VO _2_ max (ml/kg/min) of the patient groups were: 452±73 and 24.3±6.9 (coronary artery bypass grafting), 499±84 and 27.6±7.2 (aortic valve replacement), 496±85 and 27.4±7.3 (mitral valve surgery), and 519±90 and 27.3±6.9 (percutaneous coronary intervention or coronary angiography). The population-based sample had significantly greater walk test distance (623±81) and VO _2_ max (31.7±6.1) than the four patient groups (all p-values<0.001). All patient groups had lower cardiorespiratory fitness than the reference population of 60–69-year-old Finnish adults. Particularly the coronary artery bypass grafting group had a low cardiorespiratory fitness, and therefore might be prone to complications and challenging rehabilitation after the operation.

## Introduction

Globally, almost one-third of deaths are due to cardiovascular diseases (CVDs) 1 . The most frequent CVD diagnosis is coronary artery disease (CAD) 2 3 . In addition, aortic valve stenosis (AVS) and mitral valve insufficiency (MVI) constitute an increasing disease burden 3 4 . Percutaneous coronary intervention (PCI), coronary artery bypass grafting (CABG), aortic valve replacement (AVR), mitral valve replacement (MVR) and mitral valve repair (MVP) are invasive procedures of high importance to these patients 5 .

High cardiorespiratory fitness is associated with a lower CVD risk independently of other risk factors 6 7 . Low cardiorespiratory fitness is also associated with a higher risk for all-cause and CVD mortality 8 9 . In addition, poor preoperative cardiorespiratory fitness has been associated with higher mortality after CABG 10 . The 6-minute walk test (6MWT) is a simple, affordable and safe method to evaluate the functional capacity of cardiac patients 11 12 13 . Furthermore, 6MWT has also been reported to be a highly reliable means of predicting cardiorespiratory fitness in population-based samples 14 . It has been suggested that preoperative 6MWT could be a useful tool for assessment of recovery after cardiac surgery 13 15 .

In addition to cardiorespiratory fitness, physical activity (PA) is an important factor in preventing CVDs 16 , whereas sedentary behavior (SB) is a risk factor for CVDs 17 . Decreased PA is associated with complications after elective cardiac surgery 18 . Furthermore, SB may contribute to mortality after elective cardiac surgery 19 . On the other hand, accelerometer-based information about PA and SB in secondary prevention of CVDs is scarce.

Low peak exercise oxygen consumption is concluded to be a very powerful predictor of future fatal cardiac events, additionally to many conventional risk factors 20 . Further, a recent meta-analysis suggests that exercise-based coronary rehabilitation reduces mortality, cardiac events and hospitalizations, and improves quality of life 21 . Therefore, both preoperative cardiorespiratory fitness and exercise-based rehabilitation are clinically important predictors of postoperative complications and physical functioning. In this study, we hypothesized that patients scheduled for elective open-heart surgery (CABG, AVR or mitral valve surgery (MVS)) have lower cardiorespiratory fitness than patients scheduled for elective non-surgical cardiac procedures (PCI or coronary angiography (PCI-CA)). We evaluated cardiorespiratory fitness using 6MWT in patients scheduled for elective PCI-CA, CABG, AVR or MVS, and further compared the results with those measured in general age-matched Finnish reference population. In addition, correlations between 6MWT distance and different parameters of previously reported accelerometer-measured PA and SB of the patients were calculated for the first time in these cardiac patients using measured values of both fitness and activity with a great number of participants 22 .

## Materials and Methods

### Participants

The current study is based on the baseline measurements of the ongoing trial “Personalized intervention to increase physical activity and reduce sedentary behaviour in rehabilitation after cardiac operations” (PACO) 23 . The data we used in this study were collected from May 2018 until fall 2022. Patients were scheduled for elective open-heart surgery [i. e. coronary artery bypass surgery (CABG), aortic valve replacement (AVR), and mitral valve surgery (MVS)] or coronary angiography (CA) at Kuopio university hospital. Patients also met the inclusion criteria: 1) participating in the scheduled cardiac operation above; 2) willing to wear an accelerometer, and 3) willing and capable of using a smartphone app, if randomized to the intervention group for the PACO trial. Additionally, patients showed no exclusion criteria for the PACO trial. Exclusion criteria included: 1) no severe disease or functional reasons limiting PA (other than CVD); 2) patient ends up in prolonged intensive care; 3) surgery type changes during the operation; 4) patient has a memory disorder; or 5) patient does not use accelerometer as instructed. Patients who met the inclusion criteria were contacted and asked whether they would be willing to participate in the trial. If patients showed interest, they were sent the patient information sheet and informed consent form (ICF) with return envelope. Patients were also advised to contact the study personnel for additional information, if necessary. Once the patient had signed and returned the ICF, the baseline measurements were activated (see below).

For the baseline measurements, patients carried a triaxial accelerometer 24/7 for measurements of PA and SB (described in detail below). Patients returned the accelerometer upon arrival at the hospital for the scheduled cardiac operation. 6MWT was performed after admission to the hospital on the first preoperative day and supervised by a study nurse (in details below). The final allocation to the study groups was performed after the cardiac scheduled operation. For example, in some cases, patients scheduled for CABG who showed more severe mitral valve regurgitation than expected were switched to combined CABG and MVS, and reallocated into MVS group. Correspondingly, in some patients, CA indicated percutaneous coronary intervention (PCI). In most patients, PCI was performed ad-hoc (immediately following the angiography). Patients undergoing coronary angiography and PCI were combined into PCI-CA group 19 22 .

### Reference group for population-based sample

The population-based sample of Finnish adults of the FinFit2017 study was used for comparison with the cardiac procedure patients 24 . From that study, two 10-year age group and sex matched reference subjects were drawn for each patient. For patients over 69 years old, matched reference subjects were drawn from the FinFit2017 60–69 age group. The FinFit2017 study was selected, as it includes the same 6MWT and 24/7 accelerometer measurements, the collection of the data was done almost at the same time as in this study, and the FinFit2017 sample serves well as a sample of the general population of Finnish adults 24 .

### The 6-minute walk test

The cardiorespiratory fitness of the patients was evaluated preoperatively with 6MWT during the index hospitalization. 6MWT was done using the protocol instructed by the American Thoracic Society (ATS) 25 . Blood pressure (Omron M6, Omron Healthcare Co, Kyoto, Kapan) was measured at baseline, immediately after 6MWT and after 3-minute recovery. Heart rate (Polar M430, Polar Electro Ltd. Kempele, Finland) was recorded at baseline, during maximal heart rate, after 1-minute and 3-minute recovery, as well as the heart rate during possible angina pectoris. The 6MWT was conducted using a 30-meter indoor track in the corridor of the study hospital. The walking distance was measured, and if the 6MWT was interrupted, the time elapsed, distance walked and the reason why the test was interrupted were recorded. If the patient complained of angina pectoris, the time and heart rate when it occurred, together with angina severity, were recorded. Basically, a similar protocol was used in the FinFit 2017 study.

Based on 6MWT, the maximal oxygen consumption (VO _2_ max, ml/kg/min) was estimated in men from walking distance, age, BMI, heart rate at the end of test and height, and in women, from walking distance, age, and weight 14 . Ten patients discontinued the 6MWT: five because of chest pain, two feeling out of breath, one facing tiredness of legs, and two feeling dizziness.

### Accelerometer measurements

The patients wore a triaxial accelerometer (UKK RM42, UKK Terveyspalvelut Oy, Tampere, Finland) on their right hip while awake and on their wrist while sleeping 23 . The accelerometer was used for one week during the month before the scheduled cardiac procedure but was advised not to be used during any exposure to water. Instructions for the correct use of the device were provided both orally and in writing. The criterion for sufficient accelerometer carrying was 24 hours for at least four days 22 . The raw data collected by the accelerometer were stored on a hard drive for further analyses.

In 6-second epochs, the resultant acceleration (vector sum of three orthogonal components) was calculated to determine the mean amplitude deviation (MAD). As MAD values accurately predict VO _2_ consumption, they were converted into metabolic equivalents (MET, 3.5 mL/kg/min of oxygen consumption) 26 27 . Using 6-second epochs, the one-minute exponential moving average of MET values was calculated.

PA (corresponding to movement-related energy expenditure>1.5 METs) was classified according to the MET levels as follows: light (LPA, 1.5–2.9 METs) and moderate-to-vigorous (MVPA,≥3.0 METs) 22 28 . Moreover, SB (energy expenditure≤1.5 METs in sitting or reclined position) and standing (energy expenditure≤1.5 METs in upright position) were identified using the angle for posture estimation (APE) algorithm 29 30 . The parameters of PA, SB and standing, reported in a previous study 22 , were investigated for their possible correlations with 6MWT result.

Based on our earlier studies 31 and the interview executed by the study nurse, the majority of patients do not report regular physical activity. Therefore, we measured physical activity using an accelerometer. We also know that the majority of the physical activity is accumulated from walking different bout lengths. In addition, some patients bicycle during summer and some do cross-country skiing during winter, but the amount and intensity of the physical activity is generally quite low in these patient groups.

### Ethics

The ethical approval of this study was received from The Research Ethics Committee of the Northern Savo Hospital District (304/2017). Prior to the participation, a written informed consent was signed by all patients.

Regarding the FinFit2017 study, the ethical approval was received from The Regional Ethics Committee of the Expert Responsibility Area of Tampere University Hospital (R17030). A written informed consent was also signed by the participants before their participation.

### Statistical analysis

Characteristics of the patients are shown as means with standard deviations for numerical variables and counts with percentages for categorical variables. For demographics, clinical characteristics and medications, the Kruskall-Wallis test for numerical variables and Fisher’s Exact test for categorical variables were used to test the differences between patient groups. A general linear model (GLM) multivariate analysis of variance was used to test the differences in fitness tests and accelerometer variables between patient groups. The Sidak adjustment for p-values and confidence intervals was used to account for multiple comparisons between patient groups. Spearman’s rank correlation coefficient was used for correlation between 6MWT and accelerometer variables. An independent samples t-test, assuming that variances are not equal, was used to test the differences between patients and FinFit2017 reference subjects. Fisher’s Exact tests were conducted in R (R Core Team, 2020) and other analyses in SPSS 28 (IBM Corp. 2020, Armonk, NY).

## Results

**Fig. 1:**
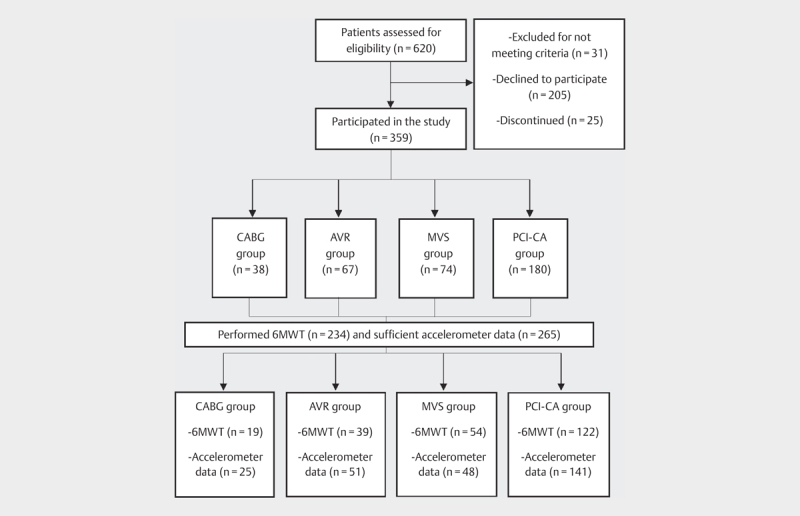
Study participation flow diagram. Abbreviations: CABG: coronary artery bypass grafting; AVR: aortic valve replacement; MVS: mitral valve surgery; PCI-CA: percutaneous coronary intervention or coronary angiography; 6MWT: six-minute walk test.

A total of 620 patients were invited, and 359 patients participated in the trial ( Fig. 1 ). The group sizes per cardiac procedure were: 1) PCI-CA (n=180); 2) CABG (n=38); 3) AVR (n=67); and 4) MVS (n=74). Of those, 234 performed 6MWT (PCI-CA: 122; CABG: 19; AVR: 39; MVS: 54). Accelerometer data were sufficient in 265 patients (PCI-CA: 141; CABG: 25; AVR: 51; MVS: 48). The demographics, clinical characteristics, and medications are presented in Table 1 . The mean age of all patients was 63.9 years (SD±9.4), and 257 (71.6%) were men.

**Table:** **Table 1** Characteristics of the patients.

	CABG (n=38)	AVR (n=67)	MVS (n=74)	PCI-CA (n=180)	p-value
Age (y)	65.1 (7.2)	61.5 (12.4)	60.0 (11.7)	66.1 (6.3)	<0.001
Male†	25 (65.8)	51 (76.1)	64 (86.5)	117 (65.0)	0.003
BMI (kg/m ^2^ )	29.2 (4.5)	28.1 (4.9)	26.6 (4.4)	27.3 (4.0)	0.034
Total cholesterol (mmol/l)	3.6 (0.8)	4.0 (1.0)	4.3 (0.9)	3.8 (1.0)	0.038
HDL cholesterol (mmol/l)	1.44 (0.42)	1.50 (0.30)	1.50 (0.27)	1.48 (0.51)	0.41
LDL cholesterol (mmol/l)	1.99 (0.77)	2.40 (0.88)	2.65 (0.94)	2.07 (0.85)	0.002
Triglycerides (mmol/l)	1.09 (0.36)	1.10 (0.77)	1.15 (0.47)	1.27 (0.72)	0.21
Smoking†	1 (3.0)	1 (1.7)	1 (1.5)	6 (4.3)	0.82
Diabetes†	17 (51.5)	10 (16.9)	2 (3.1)	28 (19.9)	<0.001
Hypertension†	30 (90.9)	34 (57.6)	26 (41.3)	93 (67.4)	<0.001
Hypercholesterolemia†	30 (90.9)	40 (70.2)	41 (66.1)	133 (95.0)	<0.001
Atrial fibrillation†	4 (13.3)	13 (23.2)	16 (25.0)	15 (11.3)	0.142
Heart failure†	0	2 (3.4)	2 (3.1)	1 (0.7)	0.31
Coronary artery disease†	33 (100)	17 (29.3)	17 (26.2)	95 (70.9)	<0.001
Arteriosclerosis obliterans†	2 (6.1)	0	0	1 (0.7)	0.080
Stroke or transient ischemic attack†	3 (9.1)	4 (6.8)	3 (4.6)	8 (5.8)	0.78
Myocardial infarction†	6 (18.2)	2 (3.4)	0	14 (10.1)	0.001
Previous percutaneous coronary intervention†	9 (27.3)	6 (10.2)	3 (4.6)	28 (20.0)	0.003
Previous CABG†	0	0	0	6 (4.3)	0.12
Previous valve surgery†	0	4 (6.8)	2 (3.1)	3 (2.1)	0.31
Pacemaker†	1 (3.0)	2 (3.4)	1 (1.5)	1 (0.7)	0.34
Lung disease†	7 (21.2)	9 (15.3)	8 (12.3)	21 (15.1)	0.71
Cancer†	0	1 (1.7)	3 (4.6)	11 (7.9)	0.19
Thyroid gland disease†	3 (9.1)	2 (3.4)	2 (3.1)	22 (15.8)	0.007
LVEF (%)	59.6 (9.0)	57.8 (9.4)	64.2 (10.2)	61.0 (7.9)	0.003
Medication†					
Beta blocker	21 (63.6)	22 (37.3)	23 (35.4)	73 (51.8)	0.014
Calcium blocker	14 (42.4)	17 (28.8)	12 (18.8)	33 (23.4)	0.075
ACE inhibitor/Angiotensin receptor blocker	23 (69.7)	33 (55.9)	28 (43.1)	77 (54.6)	0.090
Acetylsalicylic acid	25 (75.8)	23 (39.0)	16 (25.0)	95 (67.4)	<0.001
Adenosine-diphosphate receptor antagonists	5 (15.2)	3 (5.1)	2 (3.2)	6 (4.3)	0.11
Warfarin	1 (3.0)	6 (10.3)	5 (7.8)	8 (5.7)	0.55
Novel oral anticoagulant	4 (12.1)	5 (8.5)	11 (17.2)	8 (5.7)	0.068
Statin	30 (90.9)	38 (64.4)	34 (53.1)	115 (81.6)	<0.001
Ezetimibe	12 (36.4)	4 (6.8)	4 (6.3)	22 (15.6)	<0.001
Nitrate	30 (90.9)	38 (64.4)	34 (53.1)	115 (81.6)	<0.001

Mean (standard deviation) or number (percentage)†. Kruskall-Wallis test was used to analyse group differences for numerical variables and Fisher’s Exact test for categorical variables. Abbreviations: CABG: coronary artery bypass grafting; AVR: aortic valve replacement; MVS: mitral valve surgery; PCI-CA: percutaneous coronary intervention or coronary angiography; BMI: body mass index; HDL: high density lipoprotein; LDL: low density lipoprotein; LVEF: Left ventricular ejection fraction; ACE: angiotensin-converting enzyme.

**Fig. 2:**
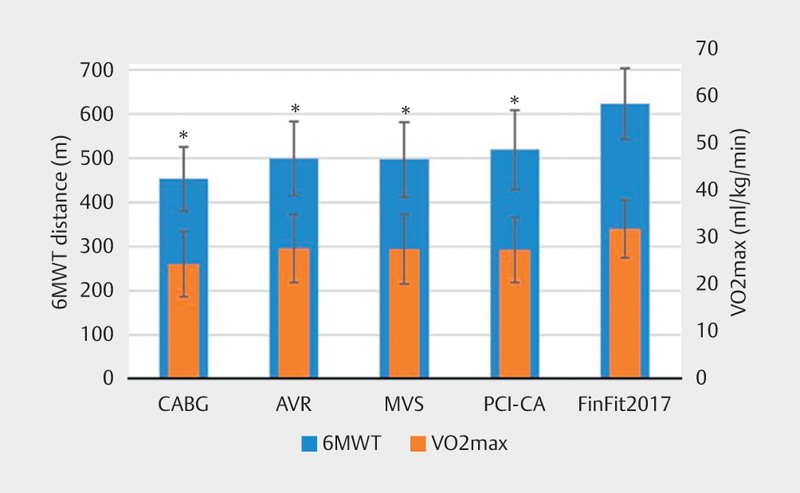
6MWT distance and VO2max of cardiac procedure patients and FinFit2017 reference population (mean and SD). *Indicates statistically significant difference (p<0.001) in both 6MWT distance and VO _2_ max, FinFit2017 as reference group. (Independent samples t-test assuming that variances are not equal.) Abbreviations: 6MWT: six-minute walk test; VO2max; maximal oxygen consumption; SD: standard deviation; CABG: coronary artery bypass grafting; AVR: aortic valve replacement; MVS: mitral valve surgery; PCI-CA: percutaneous coronary intervention or coronary angiography; FinFit2017: population-based sample of 60–69-year-old Finnish adults.

The mean (±SD) 6MWT distances (meters) in the patient groups were CABG: 452±73, AVR: 499±84, MVS: 496±85 and PCI-CA: 519±90 ( Fig. 2 ). With respect to the differences between the patient groups, the 6MWT distance in the CABG group was significantly shorter compared to the PCI-CA group (p=0.001). This difference remained significant after the Sidak adjustment (p=0.008). The four patient groups, separately and combined, had significantly shorter mean 6MWT distances than the FinFit2017 population (623±81) (all p-values<0.001).

The mean (±SD) VO _2_ max (ml/kg/min) of the four patient groups were CABG: 24.3±6.9, AVR: 27.6±7.2, MVS: 27.4±7.3 and PCI-CA: 27.3±6.9 ( Fig. 2 ). The FinFit2017 population had significantly higher mean VO _2_ max (31.7±6.1) than any of the patient groups, and all cardiac patients combined (all p-values<0.001). None of the VO _2_ max differences between patient groups were statistically significant.

The diastolic blood pressures at baseline and maximal heart rates during 6MWT were significantly lower in all patient groups compared to the FinFit2017 reference population (all p-values<0.001) ( Table 2 ). Additional information on Table 2 is available in the **supplementary material** .

**Table:** **Table 2** Blood pressures and heart rates (mean and SD) of cardiac procedure patients and FinFit2017 reference population during 6MWT.

	CABG (n=19)		AVR (n=36–38)		MVS (n=49–53)		PCI-CA (n=115–122)		FinFit2017 (n=468)	
	Mean	SD	Mean	SD	Mean	SD	Mean	SD	Mean	SD
Baseline SBP	134.3	14.5	140.4	16.2	140.0	13.6	142.5*	15.6	138.2	16.3
Baseline DBP	77.1*	12.4	74.8*	12.3	82.2*	9.9	81.5*	9.3	86.5	10.3
Max SBP	153.9	20.4	150.3	18.6	153.5	18.2	157.5	21.4		
Max DBP	78.1	13.3	76.8	13.1	83.2	10.7	83.7	11.6		
Baseline HR	68.5	11.3	73.0	13.3	71.9	13.3	71.6	15.1		
Max HR	96.9*	12.7	102.1*	15.9	101.3*	16.2	105.1*	15.6	141.1	23.5

^*^ Indicates statistically significant difference (p<0.05), FinFit2017 as reference group. (Independent samples t-test assuming that variances are not equal.). Abbreviations: SD: standard deviation; 6MWT: six-minute walk test; CABG: coronary artery bypass grafting; AVR: aortic valve replacement; MVS: mitral valve surgery; PCI-CA: percutaneous coronary intervention or coronary angiography; FinFit2017: population-based sample of 60–69-year-old Finnish adults; SBP: systolic blood pressure; DBP: diastolic blood pressure; HR: heart rate.

The correlations between 6MWT distance and different parameters of PA, standing and SB, among all cardiac patients, are presented in Table 3 . The mean daily accumulated MVPA time (r=0.418, p<0.001), the mean number of daily steps (r=0.417, p<0.001) and the mean daily accumulated time of MVPA bouts lasting<5 min (r=0.376, p<0.001) yielded the strongest positive correlations with 6MWT distance. The mean daily accumulated SB time (r=–0.283, p<0.001) and the mean daily accumulated time of SB bouts lasting 20–60 min (r=–0.248, p<0.001) yielded the strongest negative correlations with 6MWT distance. The mean daily accumulated time of physical activity, sedentary behavior, standing and time in bed among cardiac patients and the FinFit2017 population can be seen in **Supplementary Figure1** . Daily steps and accumulation of SB and MVPA from different bout lengths among cardiac patients are presented in **Supplementary Table1** .

**Table:** **Table 3** Correlations between 6MWT distance and parameters of PA, standing and SB per day among cardiac procedure patients (n=189).

	Correlation coefficient (r)	p-value
Steps (number)	0.417	<0.001
MVPA (min)	0.418	<0.001
LPA (min)	0.203	0.005
Standing (min)	0.074	0.31
SB (min)	−0.283	<0.001
<5 min MVPA bouts (min)	0.376	<0.001
5–10 min MVPA bouts (min)	0.236	0.001
>10 min MVPA bouts (min)	0.282	<0.001
<20 min SB bouts (min)	-0.039	0.60
20–60 min SB bouts (min)	-0.248	<0.001
>60 min SB bouts (min)	-0.099	0.17

The correlations were measured using Spearman’s Rank correlation coefficient. Abbreviations: 6MWT: six-minute walk test; PA: physical activity; SB: sedentary behaviour; MVPA: moderate-to-vigorous physical activity; LPA: light physical activity.

## Discussion

To our knowledge, this study is the first one to investigate the cardiorespiratory fitness of patients scheduled for elective CABG, AVR, MVS or PCI-CA, and to compare it with a population-based sample of Finnish adults. The study showed that patients who were scheduled for elective CABG had the shortest 6MWT distance of the four patient groups. In addition, all four patient groups separately and combined, had markedly shorter 6MWT distances and lower VO _2_ max values than the population-based sample of 60–69-year-old Finnish adults. For example, the FinFit2017 population sample had a 30% higher VO _2_ max than the CABG group.

In a previous study of the PACO trial 22 , we found that CABG patients had the worst activity profile of the same four patient groups, which is in line with the present results. These results suggest that CABG patients are in a vulnerable position regarding their cardiorespiratory fitness and daily activity profiles, as both potentially influence the recovery after the surgery 10 18 . CABG patients had lower cardiorespiratory fitness than PCI-CA patients, which is potentially attributable to a more diffuse CAD. Of note, the CABG and PCI-CA groups did not differ with respect to age or sex, i. e. factors known to influence exercise capacity. The cardiorespiratory fitness in both the AVR and MVS groups was quite similar to that of PCI-CA, which is in line with PA and SB levels from the previous PACO trial results 22 .

A recent study by Steinmetz et al. (2020) reported that an exercise-based preoperative intervention among CABG patients can increase 6MWT distance both pre- and postoperatively 32 . In that study, the preoperative 6MWT distances were 443 meters in the intervention group and 446 meters in the control group, which are close to the mean result of 452 m among the CABG group in the present study. Therefore, these patients should be encouraged to exercise and increase their PA. A previous exploratory study suggested that preoperative rehabilitation, also known as prehabilitation, for frail patients undergoing CABG or valve surgery might reduce the length of hospital stays 33 .

The differences in 6MWT distances and VO _2_ max values between the FinFit2017 population and cardiac patients, both in groups and combined, were large. Additionally, maximal heart rate during 6MWT was about 40 bpm higher in FinFit2017 group than in any patient group. Medication (e. g. beta blockers) may explain some differences between cardiac patients and FinFit2017 reference population, especially regarding blood pressures and heart rates. A prior study reported that a previous CABG or valve surgery were strongly associated with reduced exercise capacity in elderly CAD patients 34 . This indicates that cardiac patients’ already worse cardiorespiratory fitness compared to other population is likely to deteriorate even further after these procedures. Therefore, it is important to identify the patients with low cardiorespiratory fitness before the procedure, so that they can be a target for rehabilitation either pre- or postoperatively. The postoperative rehabilitation program could be tailored according to the patients’ preoperative functional capacity 15 .

The mean daily accumulated MVPA time and the mean number of daily steps were the parameters of PA and SB that yielded the highest positive correlations with 6MWT distance among the cardiac patients, whereas the mean daily accumulated SB time yielded the highest negative correlation. These results are in line with a study by Vaara et al. (2020) reporting that MVPA and SB time are associated with cardiorespiratory fitness among healthy, young men 35 . Additionally, in a previous study, participants with a higher cardiorespiratory fitness accumulated the most MVPA among a subsample of FinFit2017 36 . Moreover, cardiorespiratory fitness, total daily MVPA and different MVPA bouts have been shown to associate with lower Framingham CVD risk score, whereas total daily SB and different bouts of SB have a positive association with CVD risk 31 .

It is notable that the mean daily accumulated time of MVPA bouts lasting<5 min had a high correlation with 6MWT distance. This correlation was the highest regarding different MVPA bout lengths. Further, the cardiac patients accumulated more minutes of MVPA from bouts lasting<5 min than from longer 5–10 min or>10 min bouts 22 . These findings may indicate that the shortest bouts of MVPA are of crucial importance in cardiac patients 22 . Accordingly, only the most recent guidelines of PA have stated that also PA lasting<10 min is associated with health benefits 37 , while the previous recommendations used the threshold of 10 min for PA sessions beneficial for health 38 .

Another interesting finding was the surprisingly strong correlation of the mean daily accumulated time of SB bouts lasting 20–60 min with 6MWT compared to other SB bout lengths. In a previous study of PACO trial, we found that regarding different SB bout lengths, 20–60 min was also the one with most variation between patient groups, even though<20 min bouts accumulated longer mean time of SB 22 . Therefore, especially these 20–60 min bouts of SB could be potentially targeted with interventions, which could have a positive effect on both cardiorespiratory fitness and even postoperative recovery.

We chose 6MWT to measure the cardiorespiratory fitness, as it is a safe, simple, and commonly used test for cardiac procedure patients and patients with heart failure 25 . The measurement of PA and SB were done objectively with accelerometer for higher reliability and greater precision 39 . We combined the patients who had both CABG and valve surgery with the corresponding valve surgery groups. This method has been used previously 19 .

This study has several strengths. We had a rather large sample of cardiac procedure patients, and used universal and accurate measurements of cardiorespiratory fitness, PA, and SB. In addition, we included a large reference group of 60–69-year-old Finnish adults from the FinFit2017 study. On the other hand, we also acknowledge some limitations. The cross-sectional design of the study prevents recognizing any causative influence. In addition, the results should be interpreted cautiously, as the patient sample size per procedure group was limited.

## Conclusions and clinical implications

Our study revealed that patients scheduled for elective open-heart surgery or non-surgical cardiac procedure (CABG, AVR, MVS and PCI-CA) have poorer cardiorespiratory fitness than the population-based sample of age- and gender-matched Finnish adults. Since patients scheduled for CABG had the poorest cardiorespiratory fitness, they should be considered a target group for preoperative rehabilitation. As suggested by previous studies, preoperative cardiorespiratory fitness might influence the postoperative recovery and complications. Therefore, recognizing patients with low preoperative fitness would allow interventions to be targeted at them. For example, rehabilitation programs could be applied to increase PA and thereby cardiorespiratory fitness of cardiac patients, preoperatively as well as postoperatively. Precise information about the cardiorespiratory fitness, PA and SB allows creating individualized rehabilitation programs based on the preoperative fitness and activity of the patients.
