# Supplementary material for: Elective Cardiac Procedure Patients Have Low Preoperative Cardiorespiratory Fitness
Source: Int J Sports Med. 2023 Oct 6;45(1):63–70. doi: 10.1055/a-2161-4137 (PMC10776211; doi:10.1055/a-2161-4137)
Supplement: Supplementary file 1 — Supplementary Material [file 10-1055-a-2161-4137-9845.pdf]

Supplementary Material: Elective Cardiac Procedure Patients Have Low Preoperative Cardiorespiratory Fitness

Supplementary Text (Regarding Table 2)

There were some significant differences between patient groups in blood pressures and heart rates during 6MWT (Table 2). The PCI-CA group had a higher baseline systolic blood pressure than CABG group (p=0.025). AVR group had a lower baseline diastolic blood pressure than MVS group (p=0.002) and PCI-CA group (p=0.001). The maximal systolic blood pressure was higher in PCI-CA group than in AVR group (p=0.037). AVR group had a lower maximal diastolic blood pressure than MVS group (p=0.007) and PCI-CA group (p<0.001), and CABG group lower than PCI-CA (p=0.041). PCI-CA group had higher maximal heart rate than CABG group (p=0.036). However, after Sidak adjustments, the only statistically significant differences were lower baseline and maximal diastolic blood pressures in the AVR group compared to both MVS (baseline: p=0.013, maximal: p=0.042) and PCI-CA groups (baseline: p=0.003, maximal: p=0.002).

Supplementary Table1

Average steps and time spent in different bout lengths of MVPA and SB per day among cardiac procedure patients (n=267).

	CABG		AVR		MVS		PCI-CA	
	Mean	SD	Mean	SD	Mean	SD	Mean	SD
Steps (number)	4525	2849	5789	3193	5611	2419	5930*	3191
<5 min MVPA bouts (min)	13.6	14.8	18.7	16.7	16.5	10.2	18.6	14.6
5-10 min MVPA bouts (min)	3.9	5.8	5.2	7.3	3.2	3.4	5.0	5.5
>10 min MVPA bouts (min)	7.7	14.9	16.0	22.6	13.4	17.7	14.9	23.2
<20 min SB bouts (min)	254.9	59.3	259.7	62.3	251.4	52.7	247.7	56.0
20-60 min SB bouts (min)	261.3	71.7	232.6	76.6	222.2*	57.3	216.1*	67.9
>60 min SB bouts (min)	107.5	72.8	91.7	70.0	104.7	66.0	102.9	75.5

Abbreviations: MVPA: moderate-to-vigorous physical activity; SB: sedentary behaviour; CABG: coronary artery bypass grafting; AVR: aortic valve replacement; MVS: mitral valve surgery; PCI-CA: percutaneous coronary intervention or coronary angiography; SD: standard deviation

* Indicates statistically significant difference (p<0.05), CABG as reference group. (General linear model (GLM) multivariate analysis of variance.)

Supplementary Figure1. : Average time spent in bed and during wake time in physical activity,standing and sedentary behaviour in cardiac procedure patients and FinFit2017 population sample.

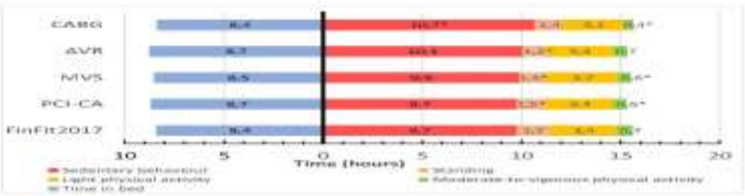

Abbreviations: CABG: coronary artery bypass grafting; AVR: aortic valve replacement; MVS: mitral valve

surgery; PCI-CA: percutaneous coronary intervention or coronary angiography; FinFit2017: populationbased
sample of 60-69-year-old Finnish adults
* Indicates statistically significant difference (p<0.05), FinFit2017 as reference group. (Independent samples
t-test assuming that variances are not equal.)

| © 2023. Thieme. All rights reserved.
